# Blindfolded Balance Training in Patients with Parkinson's Disease: A Sensory-Motor Strategy to Improve the Gait

**DOI:** 10.1155/2016/7536862

**Published:** 2016-02-09

**Authors:** M. Tramontano, S. Bonnì, A. Martino Cinnera, F. Marchetti, C. Caltagirone, G. Koch, A. Peppe

**Affiliations:** ^1^IRCCS Santa Lucia Foundation, 00179 Rome, Italy; ^2^Non-Invasive Brain Stimulation Unit, IRCCS Santa Lucia Foundation, Rome, Italy; ^3^Department of System Medicine, University of Rome Tor Vergata, 00173 Rome, Italy; ^4^Stroke Unit, Policlinico Tor Vergata, 00173 Rome, Italy

## Abstract

*Aim*. Recent evidence suggested that the use of treadmill training may improve gait parameters. Visual deprivation could engage alternative sensory strategies to control dynamic equilibrium and stabilize gait based on vestibulospinal reflexes (VSR). We aimed to investigate the efficacy of a blindfolded balance training (BBT) in the improvement of stride phase percentage reliable gait parameters in patients with Parkinson's Disease (PD) compared to patients treated with standard physical therapy (PT).* Methods*. Thirty PD patients were randomized in two groups of 15 patients, one group treated with BBT during two weeks and another group treated with standard PT during eight weeks. We evaluated gait parameters before and after BBT and PT interventions, in terms of double stance, swing, and stance phase percentage.* Results*. BBT induced an improvement of double stance phase as revealed (decreased percentage of double stance phase during the gait cycle) in comparison to PT. The other gait parameters swing and stance phase did not differ between the two groups.* Discussion*. These results support the introduction of complementary rehabilitative strategies based on sensory-motor stimulation in the traditional PD patient's rehabilitation. Further studies are needed to investigate the neurophysiological circuits and mechanism underlying clinical and motor modifications.

## 1. Introduction

Difficulty in walking is a pathognomonic sign of Parkinson's Disease (PD). Gait disorders, balance impairment, falls, and fall-related injuries are also present in PD patients [1]. Indeed, patients with PD demonstrate impaired ability to walk [2, 3] and to change direction [4]. PD patients' gait is characterized by small shuffling steps, stooped posture, and reduced arm swing. As disease progresses, these features worsen, treatment efficacy wanes, and gait impairment becomes increasingly disabling [5]. The management of PD has been traditionally based on pharmacological and surgical therapy; even with optimal medical management, PD patients experience deterioration in body function, daily activities, and participation [6]. Therefore, rehabilitation therapies represent an adjuvant to pharmacological and neurosurgical treatment [7]. The target of the traditional motor rehabilitation program was muscle stretching, motor coordination, balance, and gait trainer [8]. Recent evidence suggested that the use of treadmill training may improve gait parameters, such as gait speed and stride length [9]. Moreover, the study of kinematic alteration of the gait through gait analysis system showed specific altered spatiotemporal parameters in PD patients [10].

The gait cycle consists in three important phases of step: stance, swing, and double stance of both sides of body. These can be observed by different point of view: time, space, and jerk, but the more commune and coherent method is the normalization of step cycle in percentage (phase stride percentage) [11]. In particular, motor rehabilitation program reduced temporal variables in the stance phase and increased the swing phase; only the single support phase was decreased, while the double stance phase was not significantly changed after traditional rehabilitation program [12]. Transitioning from double stance to single stance is challenging to maintain postural stability, as one has to shift weight from a relatively stable position during double stance to a smaller base of support during single stance [13]. The relationship between altered gait and postural instability is very close in PD patients and despite optimal medication therapy, significant gait impairment remains even in very early disease [1, 14]. The impairment of sensory integration has been suggested to influence balance control in Parkinson's Disease [15]. Recent studies [16, 17] supported the role of visual deprivation as a potential driver in using alternative sensory strategies to control dynamic equilibrium and stabilize gait. Furthermore, as reported by De Nunzio et al. [18], PD patients showed central deficit in reorganizing sensory information for postural control which induces a delay in balancing strategy adaptation. These sensory processing impairments could be enhanced in PD by means of dedicated strategies during PT programs. In particular, rehabilitative training based on the enhancement of sensorial input could be essential to improve balance and gait in PD patient [19]. These assumptions indicated that more attention should be given to adopting rehabilitation strategies which improve postural responses by means of sensorial integration afferences. However, several questions remain unanswered, particularly regarding training methods as well as intensity and duration and specific exercises need to improve gait and balance control in PD. Here we introduced specific dynamic exercises performed with visual deprivation in order to stimulate reweighting of sensory information in the context of dynamic activity [20, 21]. March on foam would make inputs less reliable, so with eyes closed the subject would have to rely more on the vestibular system to maintain balance [22]. We hypothesized that rehabilitation therapy based on sensory-motor stimulation could contribute to acquisition of compensative strategies to improve gait, given the important role that the visual and proprioceptive deprivation has in sensory substitution [23]. This study aimed to investigate the efficacy of a blindfolded balance training (BBT) in the improvement of gait parameters in people with PD compared to patients who underwent physical therapy program.

## 2. Patients and Methods

Forty-four hospitalized patients with Parkinson's Disease (PD) according to the United Kingdom Parkinson's Disease Society Brain Bank clinical diagnostic criteria were enrolled in the study. Local ethical committee approved the project and written informed consents were obtained from all subjects (Prog. 297/11). Patients with (i) systemic or metabolic diseases, (ii) uncertain or unclear history of responsiveness to L-dopa treatment, (iii) presence of brain lesions or marked cortical and subcortical atrophy on brain CT and MRI scans, or (iv) dementia diagnosed by a clinical examination or a Mini-Mental State Examination score <26 [24] are excluded. The patients underwent preliminary gait analysis. Thirty subjects have been selected and were randomized with the support of Research Randomizer Software [25] in 2 groups (PT and BBT groups); each group consisted of 15 subjects. All patients were being treated exclusively with Levodopa therapy (mean: 719 mg ± 356); the pharmacological treatment was stable for at least 2 weeks before the start of the study and was not modified. Clinical data of PD patients are reported in Table 1.

### 2.1. Outcome Measures

Gait analysis was performed using the equipment and procedures developed at the motion laboratory of IRCCS Fondazione Santa Lucia, Rome, Italy. It included an optoelectronic system (SMART system, BTS, Padova, Italy) to measure the three coordinates of 23 retroreflective markers. The technical procedure is described elsewhere [12]. The Unified Parkinson's Disease Rating Scale Part III (UPDRS) [26] and gait analysis recording were carried out twice: at the beginning and at the end of rehabilitation programs (PT and BBT). All testing was carried out 2 h after the first morning's drug administration (in ON clinical status). PT group patients are tested before and after traditional rehabilitation program. BBT group patients are tested before and after blindfolded balance training program.

### 2.2. Gait Analysis

To position the markers correctly, we used an extended “Davis” protocol [27]. Extending the marker configuration of the “Davis” model, 23 spherical (10 mm in diameter) markers (axial: C7, T12, and S1; right and left: acromion, olecranon, ulnar styloid, anterior superior iliac spine, thigh, external femoral condyle, calf, external malleolus, second metatarsal head, and heel) were attached to the body with double sided tape. For the calves and thighs only, markers were attached to iron rods positioned approximately 7–10 cm away from the skin. PD subjects were blind as to when gait analysis recording would take place. The following instructions were given: “Walk as you normally do,” as reported by Jiang and Norman [28]. Gait measurements were obtained for six straight-line walking trials [11]. Patients received no additional instructions during recording and needed no physical support. The gait acquisition process involved three steps: (1) gait capture with video cameras, (2) transformation (using tracker software) of 2D acquired data into a 3D model by applying the “Davis” model, and (3) stride analysis using the extended “Davis” protocol. To perform the analysis we used “SMART” (BTS, Padova, Italy) version 1.10.427.0 software. The following variables were studied: stance, swing, and double stance percentages with respect to the stride phase [29]. The variables studied were evaluated considering (for each PD patient) the more affected body side (MABS) resulting from the clinical exam.

### 2.3. Interventions

#### 2.3.1. Physical Therapy (PT)

Therapists with experience in PD rehabilitation treated the patients individually for eight consecutive weeks; 45 min treatment sessions were held in the morning and in the afternoon five times a week. In summary, each PD patient performed 80 sessions of physical therapy (40 in the morning and 40 in afternoon). In the morning, the exercises included active and assisted limbs mobilization, four limbs coordination exercises, balance training on instable platform, gait training, and muscles stretching [30]. In the afternoon, the patients underwent a group therapy to promote control of strength, movement velocity, and motor coordination; in particular patients sitting in circle were requested to throw a ball of different size and weight to any person, increasing velocity [30].

#### 2.3.2. Blindfolded Balance Training (BBT)

Therapists with experience in PD rehabilitation treated the patients individually; for a period of two consecutive weeks 45 min treatment sessions were held in the morning in substitution of individual motor rehabilitation program and 45 min group therapy sessions were held in the afternoon five times a week. In summary, each PD patient performed 40 sessions of BBT (20 in the morning and 20 in afternoon). In the afternoon the patients of BBT group received the same treatment of PT group (control of strength, movement velocity, and motor coordination).

The BBT consisted of balance and walking exercises aimed at stimulating dynamic postural control and improving balance reactions. The main activity of the balance exercises was to march in place on a foam cushion blindfolded and walk blindfolded on a treadmill with speed increasing from 1 km/h to 3 km/h with supervision.

#### 2.3.3. March in Place

Each patient was asked to get on a foam cushion of 10 cm in height and then was blindfolded. Immediately after that he was asked to stretch his arms forward with 90° of shoulder flexion, with his hands up against the wall as a reference point. Once the position was perceived, the patient was invited not to move away ~5 cm from the wall, losing touch of hands. When the patient was in the correct position he/she was given the following instruction: “march in this position with arms extended forward for one minute.” At the end of the first minute of march, remaining blindfolded, the patient made 90° clockwise turn and repeated the exercise of marching in place for another minute. The same procedure was carried out at 180° and 270° for a total of 4 minutes. When patients made the mistake of changing direction, the physiotherapist helped them to keep the right position using verbal cues (e.g., you are turning left or right and you are going forward).

#### 2.3.4. Treadmill Training

As preparation for training, all subjects underwent a 1-minute walk on treadmill with open eyes using preferred walking speed. Immediately after preparation, patients were blindfolded and were asked to walk on treadmill without support of hands for 4 minutes. When patients made the mistake of changing direction, the physiotherapist helped them to keep the right position using verbal cues (e.g., you are turning left or right). The initial speed of the treadmill was set at 1 km/h and was increased by 0.5 km/h every minute, up to reaching a speed of 3 km/h for a total operating time of 4 minutes.

### 2.4. Statistical Analysis

One-way analyses of variance (ANOVAs) with GROUP (BBT versus PT) as between-subjects main factor were performed on baseline temporal gait parameters (stance phase, swing phase, and double stance phase). Mann-Whitney test was performed to compare UPDRS score between groups (BBT versus PT). Separate repeated-measures analysis of variance (ANOVA) was performed for the swing, stance, and double stance percentages with respect to the stride phase with GROUP (BBT versus PPT) as between-subjects main factor and TIME (before versus after) as within-subjects main factor. When a statistically significant effect was observed, Bonferroni's tests were used for post hoc analyses. For all statistical analyses, a *p* value of <0.05 was considered to be significant. Mauchly's test examined sphericity. The Greenhouse-Geisser correction was used for nonspherical data.

## 3. Results

**Figure 1:**
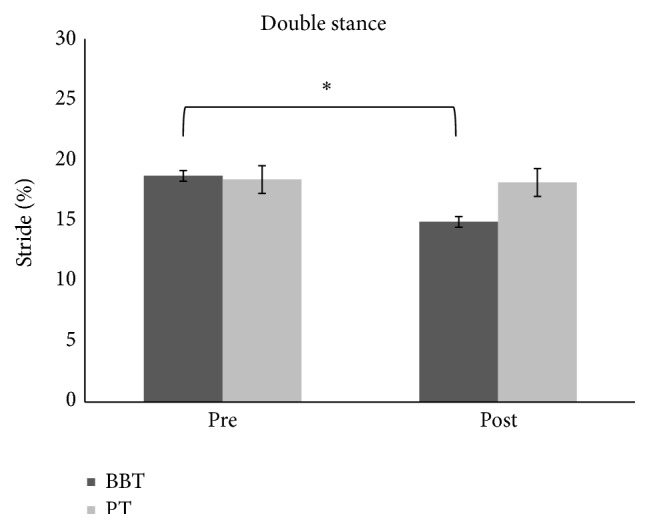
The graph shows the effects of BBT and PT (dark grey and light grey, resp.) on percentage of double stance phase with respect to entire stride phase. Error bars indicate the standard error. ^*∗*^ *p* < 0.05. PT: physical therapy; BBT: blindfolded balance training.

**Figure 2:**
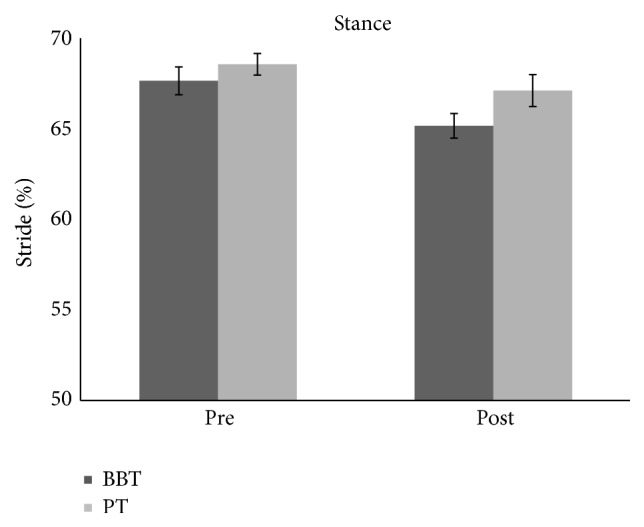
The graph shows the effects of BBT and PT (dark grey and light grey, resp.) on percentage of stance phase with respect to entire stride phase. Error bars indicate the standard error. PT: physical therapy; BBT: blindfolded balance training.

**Figure 3:**
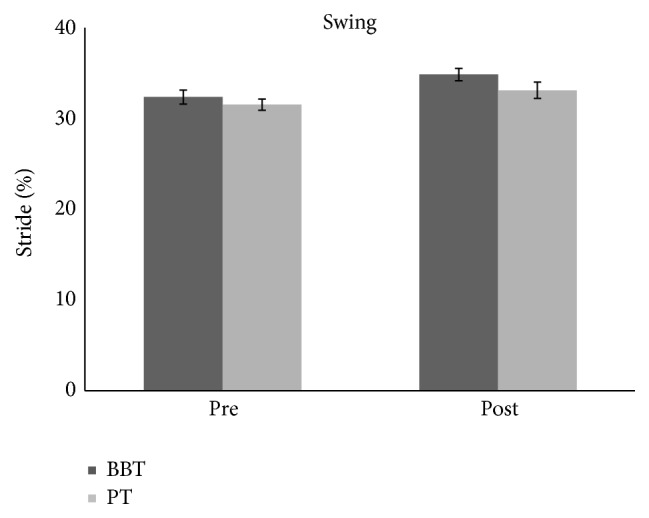
The graph shows the effects of BBT and PT (dark grey and light grey, resp.) on percentage of swing phase with respect to entire stride phase. Error bars indicate the standard error. PT: physical therapy; BBT: blindfolded balance training.

**Figure 4:**
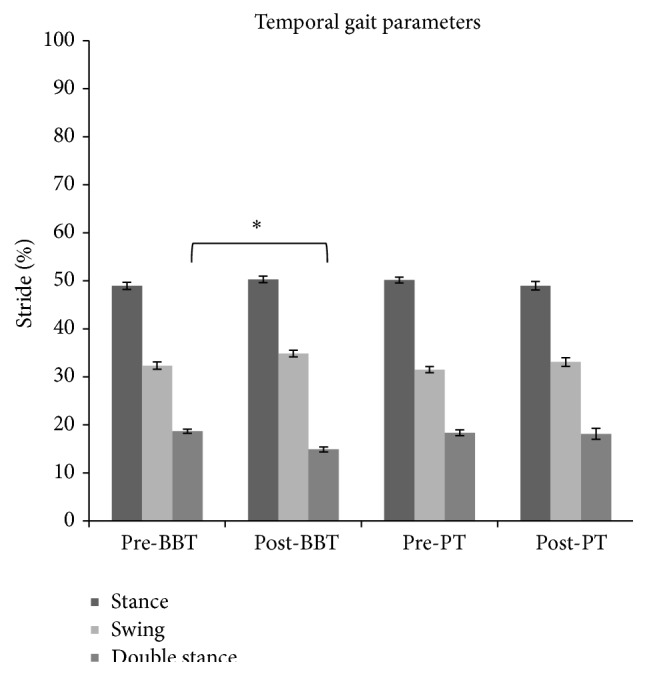
The graph shows the effects of BBT and PT on percentage of stance, swing, and double stance phases (dark grey, light grey, and grey, resp.) with respect to entire stride phase. Error bars indicate the standard error. ^*∗*^ *p* < 0.05. PT: physical therapy; BBT: blindfolded balance training.

We found no difference across groups (BBT versus PT) for baseline temporal gait parameters: stance phase (*F*(1.28) = 0.87, *p* = 0.35), swing phase (*F*(1.28) = 0.73, *p* = 0.39), and double stance phase (*F*(1.28) = 0.15, *p* = 0.69). We found no differences between groups' UPDRS scores (*p* = 0.88). BBT group has registered an improvement of double stance phase measured but with a decreased percentage in PT group, as revealed by ANOVA analysis which showed an effect of time main factor (*F*(1.28) = 12.416, *p* < 0.01) as well as GROUP × TIME interaction (*F*(1.28) = 9.55, *p* < 0.01) (Figures 1 and 4). Post hoc analysis showed that the double stance phase's percentage was significantly reduced following BBT but not PT as measured after gait analysis (*p* < 0.05). Repeated-measures ANOVA performed on the stance phase's percentage showed a main effect of TIME (*F*(1.28) = 18.02, *p* < 0.001) but no effect for GROUP main factor (*F*(1.28) = 2.3, *p* = 0.13) and for TIME × GROUP interaction (*F*(1.28) = 1.25, *p* = 0.27). Repeated-measures ANOVA performed on the swing phase's percentage showed a main effect of TIME (*F*(1.28) = 18.85, *p* < 0.001) but no effect for GROUP main factor (*F*(1.28) = 1.86, *p* = 0.18) and for TIME × GROUP interaction (*F*(1.28) = 0.93, *p* = 0.34) showing that there was a significant modulation of stance (Figures 2 and 4) and swing (Figures 3 and 4) phases' percentage in both groups following therapy but it was not specific for any type of therapy.

## 4. Discussion

This study aimed to verify the modifications of stride phase's percentage after BBT. Our results are consistent with previous finding [12] showing an increase of percentage of stance phase and decrease of swing phase's percentage in PD patients treated with physical therapy. However, we found reduction of double stance phase in PD patients treated with BBT but not with traditional rehabilitation [30]. The double stance phase's decrease is likely due to an improvement of postural stability, reflecting the patients' ability to transfer their weight correctly in preparation for stepping [13]. The double stance phase is expression of good balance control and requires the integration of sensory information from visual, somatosensory, and vestibular sources. This ability to integrate somatosensory information resulted affected in PD patients. This deficit could be compensated by the vestibular system [31–33]. Here we introduced specific dynamic exercises performed with visual deprivation coupled with gait surface changes. The treadmill induces a body acceleration that is mediated by the visual system, but to maintain the balance in visual deprivation condition the response to this acceleration should be compensated by vestibular-spinal tract. Moreover, the vestibular-spinal tract is thought to play a significant role during the execution of voluntary forward steps [34] in the double stance phase [35]. In fact vestibular information is weighted more heavily during double support than at any other time of the gait cycle [35, 36]. We hypothesize that the vestibular-spinal stimulation would contribute to the subsequent correct facilitation of Anticipatory Postural Adjustment (APA), that is, acquired motor reflexes that are necessary to perform voluntary movements. In other words, the vestibular system can primarily induce modulation of antigravitary muscles and balance reactions [21] which in turn can be learned and used by feed-forward mechanisms prior to voluntary movements.

In conclusion, our results support the hypothesis that visual deprivation and proprioceptive perturbation could be compensated using other sensory strategies as vestibular system and that this approach may be useful to improve gait in PD patients. Our findings support the introduction of complementary rehabilitative strategies based on sensory-motor stimulation in the traditional PD patient's rehabilitation program helping to achieve better functional outcomes in shorter time. Further studies are needed to verify the long term efficacy of BBT and to investigate the neurophysiological circuits and mechanism.

## Figures and Tables

**Table 1:** Clinical and demographic characteristics of Parkinson's Disease patients.

	PD patients	MABS	Age	Years of disease	UPDRS III Pre	UPDRS III Post
BBT	15	7 R	70.1 ± 8.5	7.9 ± 5.0	27.3 ± 11.4	17.8 ± 4.8
		8 L				

PT	15	7 R	69.0 ± 10.3	8.8 ± 6.6	31.2 ± 10.8	19 ± 10.54
		8 L				

PT: physical therapy; BBT: blindfolded balance training; UPDRS: Unified Parkinson's Disease Rating Scale Part III before rehabilitation treatment; MABS: more affected body side; R: right; L: left.
